# COVID-19-Related Left-Sided Ptosis

**DOI:** 10.7759/cureus.41574

**Published:** 2023-07-08

**Authors:** Dedeepya Gullapalli, Avinash Vangara, Sandhya Kolagatla, Natalia Gorrondona, Jessica Moon, Subramanya Shyam Ganti, Jayaramakrishna Depa

**Affiliations:** 1 Internal Medicine, Appalachian Regional Healthcare, Harlan, USA; 2 Internal Medicine, Appalachian Regional Healthcare, Whitesburg, USA; 3 Internal Medicine, Lincoln Memorial University, Harrogate, USA; 4 Internal Medicine/Pulmonary Critical Care, Appalachian Regional Healthcare, Harlan, USA; 5 Nephrology, Appalachian Regional Healthcare, Harlan, USA

**Keywords:** coronavirus disease, pyridostigmine, remdesivir, ace receptors, ptosis, covid-19

## Abstract

The coronavirus disease 2019 (COVID-19) infection commonly presents with symptoms of fever, cough, and anosmia. However, there have been case reports of unusual symptoms associated with COVID-19. We encountered one such case where a 55-year-old male who tested positive for COVID-19 was noted to have, along with cough and vomiting, a new onset of left eyelid ptosis. COVID-19 infection and ptosis association is seldom seen and very few similar studies are reported.

## Introduction

The coronavirus disease 2019 (COVID-19) infection displays a myriad of symptoms and signs, the most common of which are fever, chills, myalgia, headache, cough, vomiting, and diarrhea. Association of COVID-19 infection with ptosis is seldom reported. We are presenting one such case where we have observed COVID-19 infection associated with ptosis presentation.

## Case presentation

A 55-year-old male with a past medical history of hypertension and gastroesophageal reflux disease (GERD) presented to the pulmonology clinic with a complaint of persistent dry cough, body aches, and an episode of emesis that started two days ago. The patient denied wheezing, orthopnea, or leg swelling. A new onset drooping of the left eyelid prompted him to seek medical attention on the morning of his appointment.

The patient’s vitals revealed a temperature of 101.5 F, tachycardia of 108 beats per minute, tachypnea of 24 breaths per minute, blood pressure of 147/87 mmHg, and oxygen saturation of 95% on room air. The neurological examination was normal, apart from the weakness of the left upper eyelid. Pupils were equal and reactive to light. Extraocular muscles were intact, the movements were free, painless, and free. No anisocoria was noted. On further questioning, he denied a history of prior stroke, focal weakness, numbness, tingling, or changes in his speech. The patient was referred to the emergency department to rule out an acute stroke.

**Figure 1:**
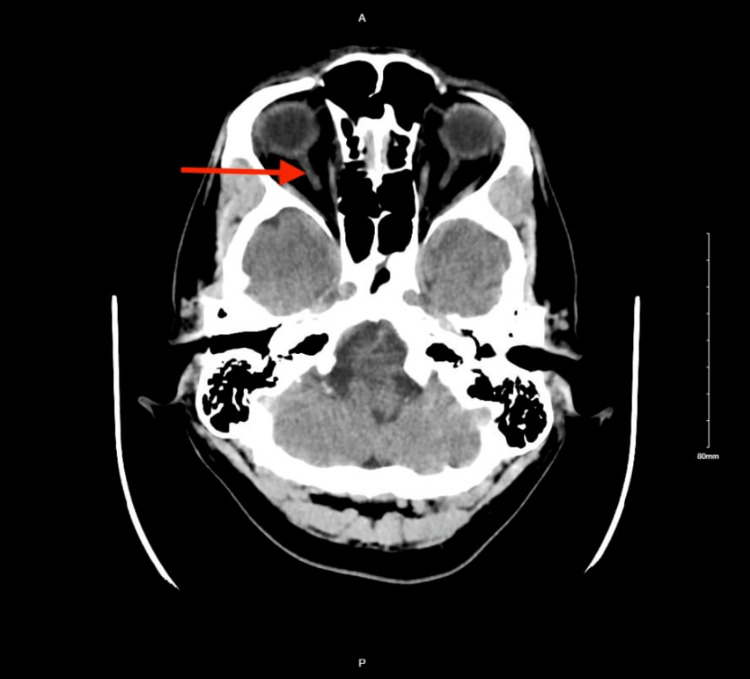
Cerebral CT with the red arrow showing the normal optic nerve

**Figure 2:**
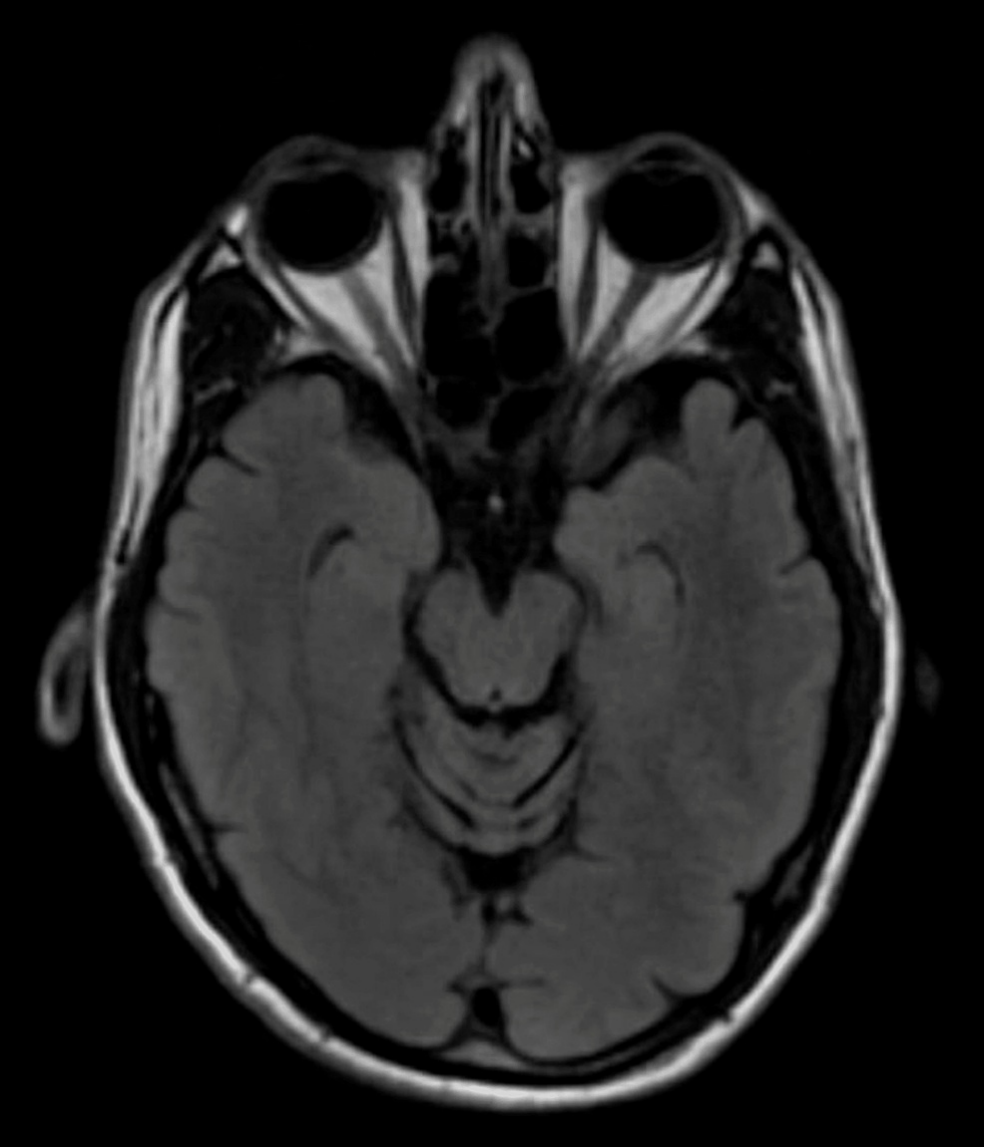
MRI of the brain showing no muscular or optic nerve edema

The patient was evaluated by neurology upon stroke alert. Head computed tomography (CT) without contrast (Figure 1) did not reveal any acute intracranial abnormalities. Head and neck CT angiography (CTA) showed no evidence of a compressive aneurysm or flow-limiting stenosis. The patient was started on aspirin and statin. Subsequent brain magnetic resonance imaging (MRI) (Figure 2) did not reveal any acute infarction.

In the ED, the rapid COVID-19 test was positive. The patient has been vaccinated with two doses of the COVID-19 vaccine without any boosters. CXR did not show radiographic evidence of acute cardiopulmonary disease.

The patient was initiated on dexamethasone 6 mg. Erythrocyte sedimentation rate (ESR) was 13 mm/r, which was found to be normal, ruling out giant cell arteritis. Later, on day one, the patient was placed on 3 L of oxygen via nasal cannula due to hypoxia, and remdesivir was initiated. He continued to receive treatment with remdesivir and dexamethasone. Further laboratory testing found his Lyme antibody value to be negative. D-Dimer levels were within normal range. Blood culture showed no growth after five days. On day two, the patient was additionally started on baricitinib 4 mg daily. On day three, the left eye ptosis was noted to have completely resolved, and the patient was discharged with a 30-day course of pyridostigmine 15 mg three times daily, considering myasthenia gravis (MG) as a differential. Later, his antibody titers came back negative, and by that time the patient's ptosis was resolved.

## Discussion

The clinical presentation and complications of COVID-19 vary widely and can affect any organ system in the body. Late complications of COVID-19 are lung fibrosis, venous and arterial thrombosis, cardiac thrombosis, inflammation, stroke, and brain fog [1]. Another well-researched complication is thrombosis, where 27% incidence of venous and 3.7% arterial thrombosis; splenic infarctions being rare among them were identified [2]. Occasionally, COVID-19 also presents with lung cavitation due to secondary bacterial infections [3]. Invasive pulmonary aspergillosis has also been reported after treatment of COVID-19 [4].

Higher morbidity and mortality rates are seen in co-infection with bacterial and viral organisms [3]. Benign neurological symptoms, like headaches and anosmia, are commonly reported with COVID-19 [5,6]. Patients with more severe infections are likely to develop neurologic manifestations (40%), especially acute cerebrovascular disease [7]. Most neurological manifestations occur in the early illness (median time being one to two days) [7].

As per our literature search, we could only find eight other cases of COVID-19-associated ptosis [8-13]. Unilateral oculomotor nerve palsy can be caused by several disorders like cerebral aneurysms, vascular conditions, tumors, or diabetes mellitus [14]. Our patient's head/neck CTA did not show any stenosis or aneurysm. The patient's glycated hemoglobin (HbA1C) is 5.6, and is prediabetic, with glucose being controlled, ruling out diabetes as a cause of neuropathy. No significant laboratory differences are noted in patients with and without peripheral nervous system (PNS) involvement [7]. Usually, inflamed oculomotor nerve palsies can be seen in the brain MRI or orbital image findings [15]. Our patient has a normal imaging and inflammatory workup for ptosis apart from positive COVID-19.

The neuroinvasive mechanism of severe acute respiratory syndrome coronavirus 2 (SARS-COV-2) is not fully understood but can be similar to how the olfactory bulb is damaged, i.e., its penetration through the riddled lamina of the ethmoid bone. This can be the route of entry to the nervous system [16]. Another mechanism is infecting the host cells via the interaction of angiotensin-converting enzyme 2 (ACE 2) receptors bound to the membrane, which is present in multiple human organs, including the nervous system, respiratory epithelium, and skeletal muscles [17]. The neurological hypothesis and ACE 2 receptor expression in the nervous system were discussed in a study done by Mao et al. on 214 cases, in which 36% of the patients had neurological symptoms [7]. It can cause direct viral injury or indirect neuroinflammatory and autoimmune mechanisms [14]. This immune response can cause swelling of the oculomotor nerve [7]. Considering how our patient did not have any signs or symptoms of other autoimmune involvement, this can be due to direct viral injury, causing inflammation of the third cranial nerve, but indirect involvement cannot be entirely ruled out.

The other explanation can be the virus directly damaging the myelin sheath and surrounding axons, like Guillain-Barré syndrome (GBS). Other ophthalmological involvement includes ophthalmoplegia with diplopia with or without headache or other signs of CNS involvement [16]. There are multiple cases of COVID-19 infection in patients with pre-existing MG, and only one case series on MG developing as a COVID-19 complication [18]. The mechanism is not clear, but the proposed theory states decreased postsynaptic acetylcholine receptors (AchRs) at the neuromuscular junction due to the destruction by the antibodies and the inflammatory response [19]. Antibodies produced by an inflammatory reaction to external agents like viruses can result in immune cross-reactivity with AchR leading to damage. The cytokines and chemokines produced from the COVID-19 infection cross-react with our own body receptors [20]. Treatment for these patients is symptomatic improvement with pyridostigmine and immunosuppressive therapy to control antibody production and decrease severity [20].

## Conclusions

Based on the findings in our case, it is essential to ask questions about weakness, decreased vision, vision loss and pain with eye movements, double vision, gait abnormalities, or other neurological conditions while screening patients with COVID-19 symptoms to rule out stroke as thrombosis risk is high with COVID-19. Physicians should also quickly assess PERRL (pupils equal, round, and reactive to light), visual acuity, pupillary response, oculomotor muscle movement, strength, and reflexes since most of these conditions can occur in the early phase of the disease. Ptosis can occur as early as the respiratory COVID-19 symptoms. Neuroimaging with angiography and stat neurology consult is crucial to rule out stroke as we did in our patient regarding morbidity and mortality.
